# An Electroanalytical Enzymeless α-Fe_2_O_3_-ZnO Hybrid Nanostructure-Based Sensor for Sensitive Quantification of Nitrite Ions

**DOI:** 10.3390/nano14080706

**Published:** 2024-04-18

**Authors:** Rafiq Ahmad, Abdullah, Md. Tabish Rehman, Mohamed F. AlAjmi, Shamshad Alam, Kiesar Sideeq Bhat, Prabhash Mishra, Byeong-Il Lee

**Affiliations:** 1 ‘New-Senior’ Oriented Smart Health Care Education Center, Pukyong National University, Busan 48513, Republic of Korea; 2 Centre for Nanoscience and Nanotechnology, Jamia Millia Islamia (Central University), Jamia Nagar, New Delhi 110025, India; 3 Future Energy Convergence Core Center, Jeonbuk National University, Jeonju 54896, Republic of Korea; abdullahazmi@jbnu.ac.kr; 4 Department of Pharmacognosy, College of Pharmacy, King Saud University, Riyadh 11451, Saudi Arabia; mrehman@ksu.edu.sa (M.T.R.); malajmii@ksu.edu.sa (M.F.A.); 5 Department of Pharmacology & Therapeutics, Roswell Park Cancer Institute, Buffalo, NY 14263, USA; shamshad.alam@roswellpark.org; 6 Department of Bioresources, University of Kashmir, Hazratbal, Srinagar 190006, India; ksb.brfellow@uok.edu.in; 7 Quantum Materials and Devices Laboratory, Faculty of Engineering and Technology, Jamia Millia Islamia (Central University), Jamia Nagar, New Delhi 110025, India; pmishra@jmi.ac.in; 8 Industry 4.0 Convergence Bionics Engineering, Pukyong National University, Busan 48513, Republic of Korea; 9 Digital Healthcare Research Center, Institute of Information Technology and Convergence, Pukyong National University, Busan 48513, Republic of Korea; 10 Division of Smart Healthcare, College of Information Technology and Convergence, Pukyong National University, Busan 48513, Republic of Korea

**Keywords:** iron oxide nanoparticle, zinc oxide nanorod, hybrid nanostructure, enzymeless, nitrite, sensitive sensor

## Abstract

Nitrite monitoring serves as a fundamental practice for protecting public health, preserving environmental quality, ensuring food safety, maintaining industrial safety standards, and optimizing agricultural practices. Although many nitrite sensing methods have been recently developed, the quantification of nitrite remains challenging due to sensitivity and selectivity limitations. In this context, we present the fabrication of enzymeless iron oxide nanoparticle-modified zinc oxide nanorod (α-Fe_2_O_3_-ZnO NR) hybrid nanostructure-based nitrite sensor fabrication. The α-Fe_2_O_3_-ZnO NR hybrid nanostructure was synthesized using a two-step hydrothermal method and characterized in detail utilizing x-ray diffraction (XRD), field emission scanning electron microscopy (FESEM), transmission electron microscopy (TEM), and X-ray photoelectron spectroscopy (XPS). These analyses confirm the successful synthesis of an α-Fe_2_O_3_-ZnO NR hybrid nanostructure, highlighting its morphology, purity, crystallinity, and elemental constituents. The α-Fe_2_O_3_-ZnO NR hybrid nanostructure was used to modify the SPCE (screen-printed carbon electrode) for enzymeless nitrite sensor fabrication. The voltammetric methods (i.e., cyclic voltammetry (CV) and differential pulse voltammetry (DPV)) were employed to explore the electrochemical characteristics of α-Fe_2_O_3_-ZnO NR/SPCE sensors for nitrite. Upon examination of the sensor’s electrochemical behavior across a range of nitrite concentrations (0 to 500 µM), it is evident that the α-Fe_2_O_3_-ZnO NR hybrid nanostructure shows an increased response with increasing nitrite concentration. The sensor demonstrates a linear response to nitrite concentrations up to 400 µM, a remarkable sensitivity of 18.10 µA µM^−1^ cm^−2^, and a notably low detection threshold of 0.16 µM. Furthermore, its exceptional selectivity, stability, and reproducibility make it an ideal tool for accurately measuring nitrite levels in serum, yielding reliable outcomes. This advancement heralds a significant step forward in the field of environmental monitoring, offering a potent solution for the precise assessment of nitrite pollution.

## 1. Introduction

Nitrite (NO_2_^−^) stands as a significant environmental and health hazard, affecting both humans and animals due to its prevalent use as a preservative in food and beverages, leading to its increased presence in water sources [1,2]. Recognizing this threat, the Environmental Protection Agency has established a maximum permissible nitrite concentration in water at 1 mg/L to safeguard public health. The consumption of excess meat/water with nitrite contamination poses health risks. Some reports indicate that an increased number of cases associated with stomach/colorectal cancer, intrauterine growth restriction, congenital defects, and methemoglobinemia were due to the consumption of nitrite-preserved meat or nitrite-contaminated water [3]. While the direct link between nitrites and cancer remains unclear, their metabolites, specifically nitrosamines, have been identified as carcinogens in various animal species [4]. Hence, nitrite detection is crucial for both therapeutic and toxicity considerations.

Various traditional nitrite detection methods such as spectrophotometry [5], chromatography [6], chemiluminescence [7], and capillary electrophoresis [8] have been employed. Most nitrite detection methods suffer from drawbacks like high cost, complex instrumentation, toxic reagent use, laborious sample preparation, and unsuitability for real-time analysis [9,10]. In contrast, electrochemical sensors emerge as a superior alternative, boasting benefits like affordability, simplicity, speed, selectivity, and high sensitivity [11]. Recent advancements have led to the creation of various nitrite sensors using nanomaterial matrices, which enhance the surface area for the effective immobilization of proteins or enzymes [11,12]. Despite the high performance of enzyme-based sensors, their limitations include cost, instability, and susceptibility to deactivation under chemical or thermal stress, making enzymeless sensing electrodes a promising solution for accurate nitrite detection [13,14,15].

Enzymeless nitrite sensing technologies leverage nanostructured materials as catalytic agents to enhance the electrochemical reaction on electrode surfaces in nitrite’s presence [10,11,16,17,18]. In this regard, an extensive effort has been made to synthesize different nanostructured materials with unique physicochemical properties of natural enzymes [16,17,18]. Such nanomaterials are known as “nanozymes”. They merge the benefits of their natural equivalents, including high stability, extensive surface area, advantages of low-cost production on a large scale, tunable catalytic activity, and straightforward modification [15]. Recently, iron-based nanomaterials have been widely used in different fields (i.e., energy storage, biomedicine, sensors, catalysis, etc.) due to their low-cost synthesis, good biocompatibility, high chemical stability, and excellent electron transfer ability [19,20,21]. Among different types of iron-based nanomaterials, hematite (α-Fe_2_O_3_), maghemite (γ-Fe_2_O_3_), and magnetite (Fe_3_O_4_) are widely used for the detection of different analytes (i.e., gases, biomolecules, organic compounds, heavy metals, ions, drugs, and pesticides) [22,23,24,25].

Due to the semiconducting properties of the α-Fe_2_O_3_ nanomaterials, they are opted for in the sensor applications [26,27,28]. These nanomaterials-based sensors offer the advantage of being highly sensitive, relatively low cost, and capable of operating in a wide range of conditions, making them suitable for the detection of these and other analytes in complex matrices. Additionally, for further improvement of the α-Fe_2_O_3_ nanomaterials-based sensors, other highly conductive nanomaterials (i.e., metal oxides, carbon nanomaterials, and conductive polymers) are often modified using iron oxide to create hybrid nanomaterials, which improve the electrochemical sensing properties of sensors [29].

The integration of α-Fe_2_O_3_ with other nanomaterials has paved the way for the production of nanocomposites that are environmentally benign yet highly effective in sensing technologies [21,23]. Among the different nanomaterials, zinc oxide (ZnO) nanostructures have been effectively modified using α-Fe_2_O_3_ nanostructures to prepare hybrid/nanocomposites [30,31,32,33,34]. The composition of α-Fe_2_O_3_ and ZnO nanostructures utilizes the synergetic effect, which enhances the performance of the hybrid nanomaterial. Additionally, ZnO nanostructures can be synthesized in large amounts using the hydrothermal method, which is a cost-effective and straightforward process [35,36]. Due to their high surface area-to-volume ratio, ZnO NRs are particularly advantageous for chemical/biological sensors, offering great promise as highly sensitive and stable sensors [35,36,37,38,39].

Innovative hybrid nanostructures-based nitrite sensors with impressive sensitivity, reliability, and longevity have been reported [40,41,42]. These advancements highlight the significant potential of iron oxide and its composites in refining the performance and environmental sustainability of nitrite sensors. Despite the great advantages of iron oxide-based hybrid nanostructures in designing nitrite sensors, there are still issues related to the synthesis of such nanostructures. The synthesis of hybrid nanomaterials in low quantities poses challenges for scaling up production, underscoring the crucial need for methodologies that can transition hybrid nanostructure synthesis from small to large scales effectively. To address this, meticulous planning, the adoption of novel synthesis approaches, and frequently, the creation of tailored analytical methods are essential. These steps ensure that the production of nanomaterials in large amounts can satisfy both research needs and industrial applications, facilitating broader utilization and innovation in sensor fabrication.

Herein, we present a two-step synthesis route for α-Fe_2_O_3_-ZnO NR hybrid nanostructure synthesis using a simple hydrothermal method. In the first step, ZnO NR was synthesized, and in the next step, the ZnO NR surface was decorated with α-Fe_2_O_3_ nanoparticles. Step two in the synthesis process can be scaled up easily to obtain a large amount of hybrid nanostructures. Using an α-Fe_2_O_3_-ZnO NR hybrid nanostructure on the working electrode of the sensor, we showcase its capability towards nitrite detection. Through electrochemical testing (using CV), we found that fabricated enzymeless α-Fe_2_O_3_-ZnO NR/SPCE nitrite sensors could achieve a linear range of up to 400 µM with remarkable sensitivity (18.10 µA µM^−1^ cm^−2^). Moreover, its unparalleled selectivity, stability, and consistent performance render it a perfect instrument for precisely determining nitrite concentrations in serum, thus ensuring dependable results.

## 2. Materials and Methods

### 2.1. Chemicals

All reagents utilized in the experiments were of analytical quality. The reagents included hexamethylenetetramine (HMTA, 99%), potassium chloride (KCl), iron(III) nitrate nonahydrate (Fe(NO_3_)_3_·9H_2_O, ≥98%), ammonium chloride (NH_4_Cl), zinc nitrate hexahydrate (Zn(NO_3_)_2_·6H_2_O, 99%), lead(II) nitrate (Pb(NO_3_)_2_), nitrite ion solution (10 mM), sodium nitrate (NaNO_3_), ethylene glycol (99.8%), sodium sulfate (Na_2_SO_4_), magnesium sulfate (MgSO_4_), calcium chloride (CaCl_2_), calcium carbonate (CaCO_3_), and PBS (phosphate buffered saline, 1.0 M, pH 7.4), all of which were sourced from Sigma-Aldrich (St. Louis, MO, USA). The preparation of all solutions was carried out using Milli-Q water.

### 2.2. α-Fe_2_O_3_-ZnO NR Hybrid Nanostructure Synthesis

**Figure 1:**
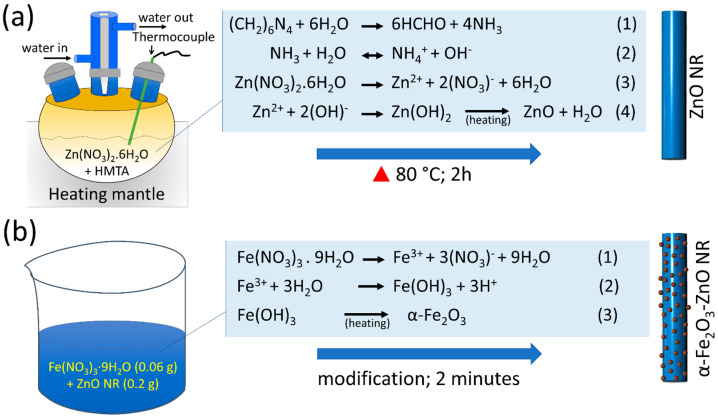
Schematic of (**a**) ZnO NR and (**b**) α-Fe_2_O_3_-ZnO NR hybrid nanostructure synthesis.

A two-step hydrothermal synthesis route was adopted to synthesize the α-Fe_2_O_3_-ZnO NR hybrid nanostructure (Figure 1). In step one, ZnO NR was synthesized in a heating mantle with a round-bottom, three-neck Pyrex glass container. The ZnO NR synthesis process scheme with chemical reactions involved is shown in Figure 1a. An equimolar solution of Zn(NO_3_)_2_·6H_2_O and HMTA (50 mM each) was prepared in the 200 mL Milli-Q water. The prepared precursor solution was transferred into a three-neck Pyrex glass container and heated for 2 h at 80 °C. Then, the solution was allowed to cool down, followed by washing in ethanol and Milli-Q water to remove impurities. The obtained ZnO NR powder was collected, dried, and annealed at 300 °C for 1 h. In step two (Figure 1b), the surface of the ZnO NR was decorated with α-Fe_2_O_3_ NPs using our previously optimized synthesis route with slight modification [40]. In brief, first, a solution of Fe(NO_3_)_3_·9H_2_O (0.06 g) was prepared in 20 mL Milli-Q water, and 0.2 g ZnO NR powder was poured into the above precursor solution and mixed for 2 min. A large amount of water was added after 2 min and centrifuged to collect the Fe(OH)_3_-ZnO NR. The Fe(OH)_3_-ZnO NR powder was dried at 40 °C, followed by annealing at 400 °C for 2 h to obtain the α-Fe_2_O_3_-ZnO NR hybrid nanostructure. The obtained α-Fe_2_O_3_-ZnO NR hybrid nanostructure was characterized in detail before its use in nitrite sensor fabrication.

### 2.3. Instrumentation

Structural and compositional properties of the α-Fe_2_O_3_-ZnO NR hybrid nanostructure were examined using X-ray diffraction (XRD) analysis performed on a Rigaku instrument, spanning a 2θ range of 20–60° (scanning rate = 8°/min). The chemical states of the α-Fe_2_O_3_-ZnO NR hybrid nanostructure were further explored through X-ray photoelectron spectroscopy (XPS) using an AXIS-NOVA device from Kratos Analytical Ltd (Manchester, UK). The morphology of the α-Fe_2_O_3_-ZnO NR hybrid nanostructure was detailed using a Carl Zeiss SUPRA 40 VP (Oberkochen, Germany) scanning electron microscope. For detailed insight into the hybrid nanostructure, transmission electron microscopy (TEM) and high-resolution (HR) TEM analyses were conducted using JEM-2010 from JEOL Ltd. (Tokyo, Japan).

### 2.4. Nitrite Sensor Fabrication and Sensing Measurements

**Figure 2:**
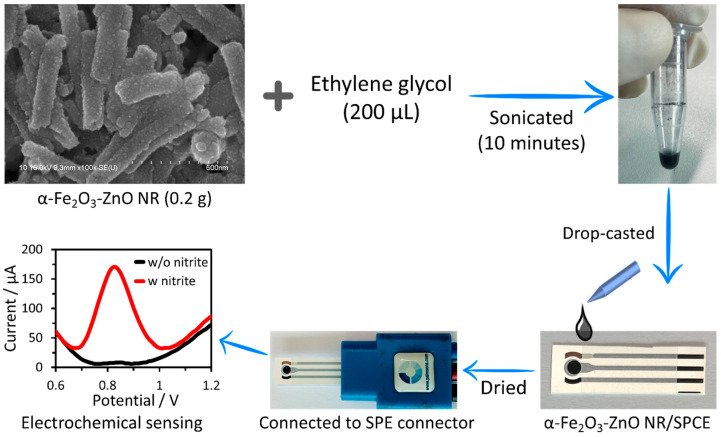
Schematic of the nitrite sensor fabrication and electrochemical sensing using nitrite sensor attachment to the SPE connector.

A cost-effective, screen-printed carbon electrode (SPCE, supplied by PalmSens (Houten, The Netherlands)) measuring 4.5 cm in length and 0.9 cm in width was utilized to construct a nitrite sensor. This SPCE comprises carbon (graphite) working (diameter = 3 mm) and counter electrodes, and is accompanied by a silver pseudo-reference electrode. The process of creating the nitrite sensor with the synthesized α-Fe_2_O_3_-ZnO NR hybrid nanostructure is illustrated in Figure 2. To begin, a dense slurry containing 200 mg of the α-Fe_2_O_3_-ZnO NR hybrid nanostructure was mixed in 200 μL of ethylene glycol and sonicated. Subsequently, to determine the optimal slurry quantity, the slurry was applied to three separate SPC electrodes in amounts ranging from 2 to 6 μL. The drop-casting technique was employed for the slurry’s application onto the electrode surface, which was then left to dry at 40 °C overnight. This step was crucial for removing the binder and ensuring that the slurry was firm. The completed nitrite sensors were then kept at room temperature, pending further evaluation.

The electrochemical properties of both bare and modified SPCEs were evaluated using a PalmSens4 device (Houten, The Netherlands), which is compact, versatile, and powerful, and was connected to a PC through Bluetooth/USB. The nitrite sensor was linked to the PalmSens4 using an SPE connector (Houten, The Netherlands). Electrochemical tests for the fabricated electrodes (both bare and modified) were initially conducted in a 10 mL probe solution containing 5.0 mM [Fe(CN)_6_]^3−/4−^ and 0.1 M KCl, employing CV at a scan rate of 50 mV/s and a potential range of −0.4 to +0.6 V. Subsequently, an optimized nitrite sensor was applied to detect nitrite in a 0.1 M PBS solution (pH 7.4) using DPV, which offers greater sensitivity than CV by reducing the capacitive current. Nitrite concentration solutions were prepared in 0.1 M PBS (pH 7.4), and a consistent potential range of +0.6 to +1.2 V was maintained for all DPV measurements.

## 3. Results

### 3.1. Material Characterization

**Figure 3:**
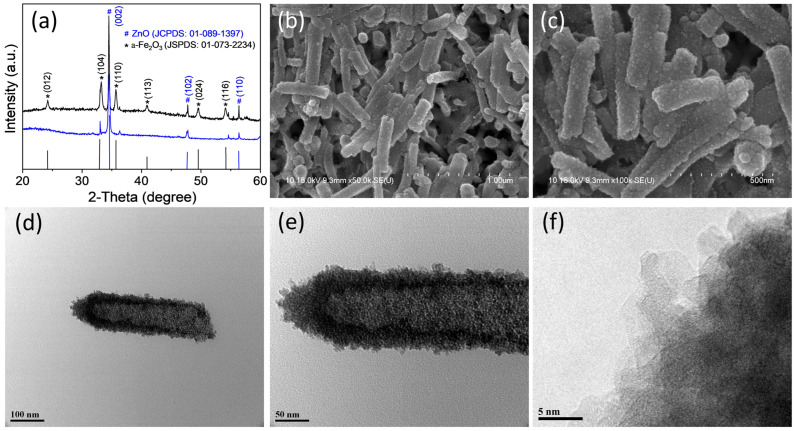
XRD spectra of ZnO NR and α-Fe_2_O_3_-ZnO NR hybrid nanostructure (**a**), FESEM (**b**,**c**), and TEM images (**d**–**f**) of the α-Fe_2_O_3_-ZnO NR hybrid nanostructure.

XRD is a paramount technique, extensively utilized across various fields for the qualitative and quantitative analysis of crystalline materials. By meticulously analyzing diffraction patterns, XRD offers insights into detailed characterizations of materials. The XRD results of the ZnO NR and α-Fe_2_O_3_-ZnO NR hybrid nanostructure are depicted in Figure 3a. The XRD pattern of ZnO NR corresponded to the hexagonal wurtzite structure of ZnO, characterized by distinctive diffraction peaks. These peaks were observed at 2θ values of 34.53° (002), 47.80° (102), and 56.35° (110), confirming the ZnO NRs’ wurtzite phase [43]. In the hybrid nanomaterials, it is notable that both materials (i.e., α-Fe_2_O_3_ nanoparticle and ZnO NR) exhibit diffraction peaks at specific angles (2θ) that correlate to distinct crystal planes. The XRD pattern shows the diffraction peaks at 2θ = 24.24° (012), 33.23° (104), 35.71° (110), 49.59° (024), and 54.10° (116), and planes are assigned to the crystal structure of iron oxide in hematite (α-Fe_2_O_3_) form [30,44]. The presence of peaks for both materials suggests the coexistence, multiphase nature, crystalline composition, and hybrid nanostructure synthesis [31].

The FESEM images were obtained to examine the surface morphology of the as-synthesized α-Fe_2_O_3_-ZnO NR hybrid nanostructure. To mitigate the charging effect during imaging, the samples were coated with a 2 nm layer of gold. The FESEM images of the as-synthesized α-Fe_2_O_3_-ZnO NR hybrid nanostructure are presented in Figure 3b,c. As expected, the hybrid nanostructure exhibits a characteristic nanorod-like morphology and rough surface. The rough surface is due to the potential incorporation of α-Fe_2_O_3_ NPs over the surface of the ZnO NR. Next, TEM was utilized to further investigate the surface modification. As shown in Figure 3d–f, the α-Fe_2_O_3_ NPs anchored on the ZnO NR are uniformly covering the ZnO surface. Furthermore, the high-resolution TEM image revealed a densely modified NR surface with α-Fe_2_O_3_ NPs (Figure 3f).

**Figure 4:**
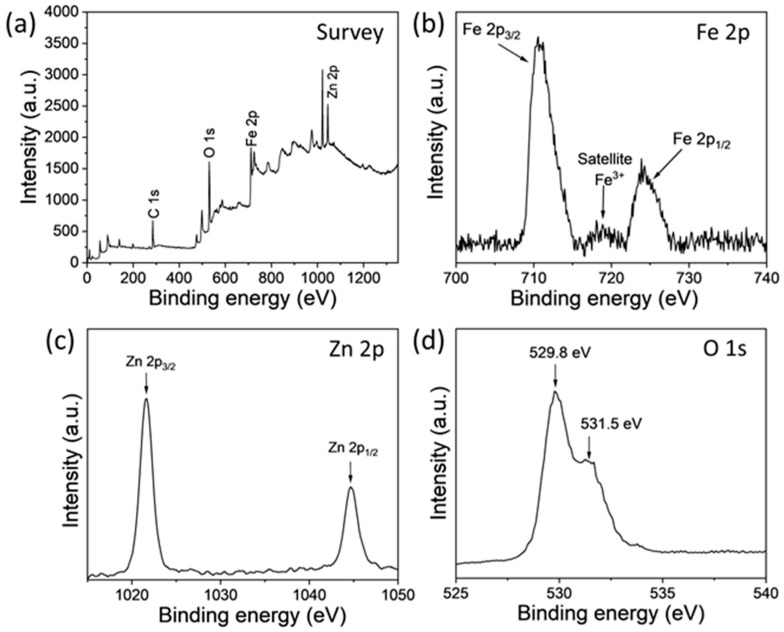
XPS wide-range spectrum survey of as-synthesized α-Fe_2_O_3_-ZnO NR hybrid nanostructure (**a**) with narrow-range spectra of Fe 2p (**b**), Zn 2p (**c**), and O 1s (**d**).

XPS analysis is critical for a comprehensive understanding of the synthesized nanomaterials. The XPS analysis was conducted to check the surface modification, chemical state, and elemental composition of the α-Fe_2_O_3_-ZnO NR hybrid nanostructure sample (Figure 4). In the wide-range survey spectrum (Figure 4a), four intense peaks signal the presence of elements, such as carbon (C 1s), oxygen (O 1s), zinc (Zn 2p), and iron (Fe 2p). The narrow-range spectra of the Fe 2p, illustrated in Figure 4b, exhibit two prominent binding energy peaks at 710.5 eV and 723.8 eV for Fe 2p_3/2_ and Fe 2p_1/2_, respectively [45]. An additional satellite peak located at 718.5 eV is indicative of Fe^3+^ ions within Fe_2_O_3_ [45]. The detailed high-resolution spectrum for Zn 2p, illustrated in Figure 4c, demonstrated two prominent peaks at 1021.6 eV (Zn 2p_3/2_) and 1044.7 eV (Zn 2p_1/2_) [46]. The difference between the two peaks (Zn 2P_1/2_-Zn 2p_3/2_) is 23.1 eV, which indicates a Zn^2+^ presence in the ZnO lattice [47]. Figure 4d shows two additional O 1s peaks at 529.8 eV and 531.5 eV in the spectrum. This is due to the presence of oxygen in the crystal lattice, specifically oxygen atoms bonded to zinc (Zn-O) and iron (Fe-O) [47]. This thorough XPS analysis unequivocally demonstrates the successful synthesis of the α-Fe_2_O_3_-ZnO NR hybrid nanostructure, highlighting the presence of elemental constituents and their associated chemical states.

### 3.2. Electrochemical Properties of α-Fe_2_O_3_-ZnO NR Hybrid Nanostructure

**Figure 5:**
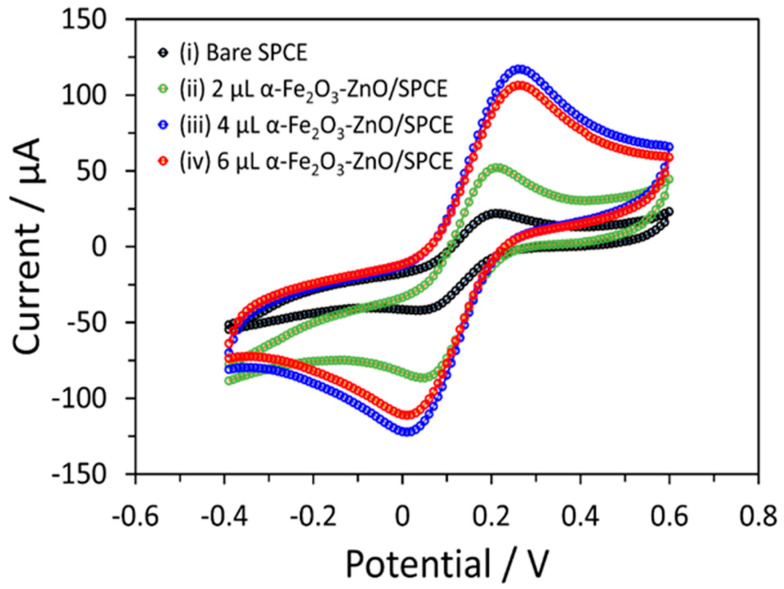
CV responses of bare and modified SPCE in 5.0 mM [Fe (CN)_6_]^3−/4−^ and 0.1 M KCl in 0.1 M PBS. The scan rate of 50 mV/s was kept the same for each electrode.

Electrochemical characteristics of bare and modified SPC electrodes were explored through CV, as detailed in Figure 5. This analysis revealed notable oxidation and reduction signals, indicative of the redox process of Fe^2+^/^3+^ on the electrode surface. Furthermore, the CV profile displayed an increase in the current response. These features are attributed to the incorporation of the α-Fe_2_O_3_-ZnO NR hybrid nanostructure onto the surface of SPCE. On the contrary, a large amount of α-Fe_2_O_3_-ZnO NR hybrid nanostructure (6 µL) modified electrodes showed a slight decrease in the current response. This might be due to the nanomaterial’s thick film over the electrode’s surface, which restricts electron transfer. Additionally, 4 µL of α-Fe_2_O_3_-ZnO NR hybrid nanostructure-modified electrodes were taken for further detailed sensing measurements.

**Figure 6:**
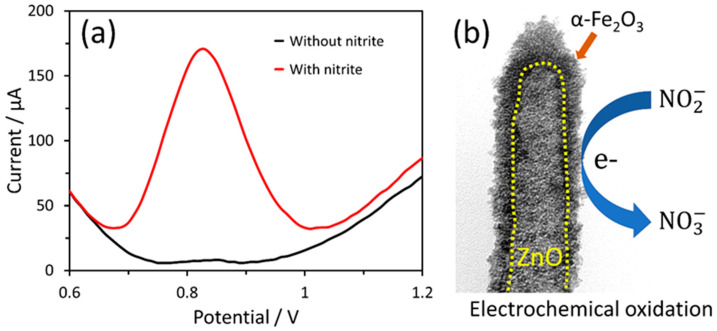
(**a**) DPV responses of the 4 µL α-Fe_2_O_3_-ZnO NR hybrid nanostructure-modified electrodes in PBS without and with 100 µM nitrite. (**b**) Schematic representation for the electrocatalytic oxidation of nitrite on the α-Fe_2_O_3_-ZnO NR hybrid nanostructure.

The catalytic efficiency of the α-Fe_2_O_3_-ZnO NR hybrid nanostructure in the oxidation of nitrite was evaluated using a 100 µM nitrite ion solution in 0.1 M PBS (Figure 6a). Initially, the solution was deoxygenated before testing to eliminate the risk of false positives arising from oxygen interference. The DPV was utilized to measure the response of the enzymeless α-Fe_2_O_3_-ZnO NR hybrid nanostructure-based nitrite sensor. When the nitrite ion was absent, the no response DPV peak was observed in PBS alone, indicating negligible oxidation activity. However, the introduction of a 100 µM nitrite ion resulted in a pronounced and sharp anodic peak, highlighting the reactive capability of α-Fe_2_O_3_-ZnO NR towards nitrite. The peak potential was identified at +0.83 V. Notably, when compared to the unmodified SPCE, α-Fe_2_O_3_-ZnO NR/SPCE demonstrated a multifold increase in the current. This significant enhancement in the current is attributed to the augmented catalytic activity brought about by the α-Fe_2_O_3_-ZnO NR modification, showcasing the potential of α-Fe_2_O_3_-ZnO NR as an effective enzymeless electrocatalyst for nitrite oxidation. During catalysis, the generated charge is transferred to the electrode surface [48]. The process underlying the electrocatalytic oxidation of nitrite on the α-Fe_2_O_3_-ZnO NR hybrid unfolds as detailed below [41].

(1) $${{Fe}_{2}O_{3}-ZnO+NO}_{2}^{-}⇌{{Fe}_{2}O_{3}-ZnO(NO}_{2}^{-})$$

(2) $${{Fe}_{2}O_{3}-ZnO(NO}_{2}^{-})⇌{Fe}_{2}O_{3}-ZnO+{NO}_{2}+e^{-}$$

(3) $${2NO}_{2}+H_{2}O⇌{2H}^{+}+{NO}_{3}^{-}+{NO}_{2}^{-}$$

(4) $${NO}_{2}^{-}+H_{2}O⇌{NO}_{3}^{-}+{2H}^{+}+{2e}^{-}$$

Equation (1) illustrates the initial interaction where nitrite forms a complex with the Fe_2_O_3_-ZnO, denoted as Fe_2_O_3_-ZnO(${NO}_{2}^{-}$). Following this formation, the complex undergoes a reaction where it releases nitrogen dioxide (NO_2_) and an electron, as depicted in Equation (2). Subsequently, nitrite and nitrate are generated through the disproportionation of NO_2_, as shown in Equation (3). The process culminates with the electrochemical oxidation of nitrite, leading to the formation of nitrate, as outlined in Equation (4). The schematic representation for the electrocatalytic oxidation of nitrite on the α-Fe_2_O_3_-ZnO NR hybrid nanostructure is shown in Figure 6b.

### 3.3. Electrochemical Determination of Nitrite Ions

**Figure 7:**
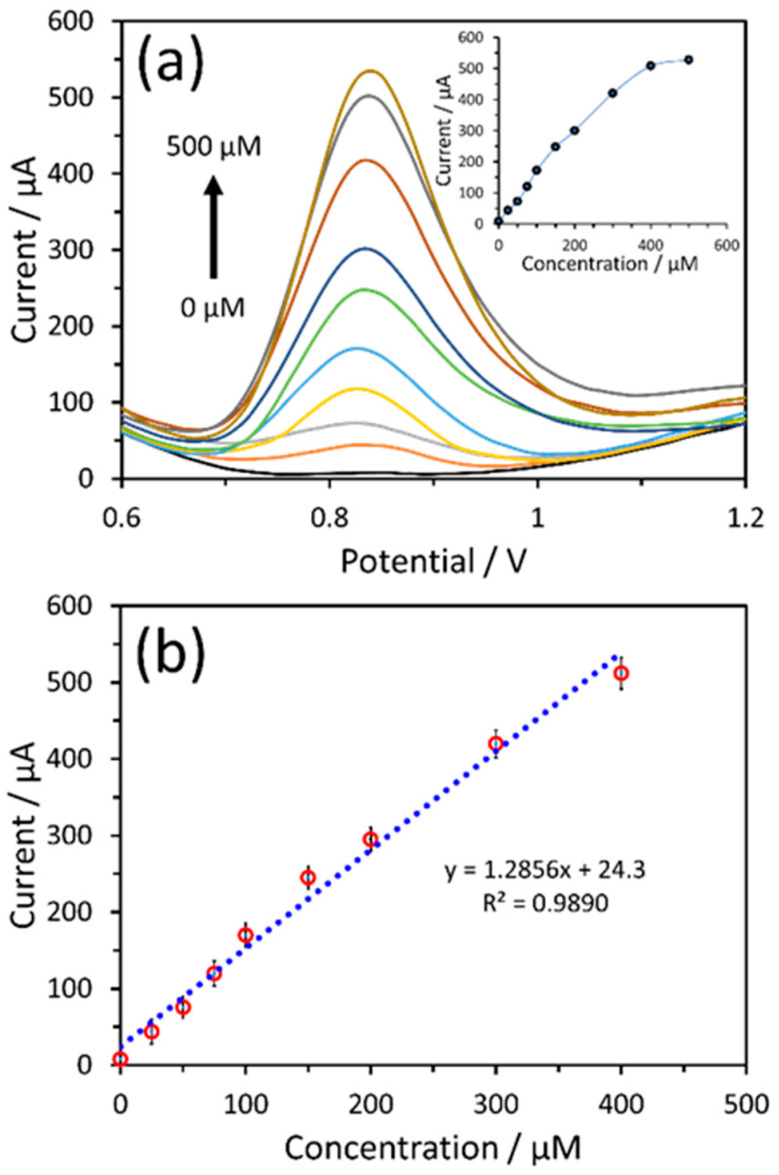
DPV responses of the 4 µL α-Fe_2_O_3_-ZnO NR hybrid nanostructure-modified electrode tested in PBS containing different concentrations of nitrite (**a**) and corresponding calibration plots of current vs. nitrite concentration (**b**). Inset (**a**) shows the plot with linear and non-linear regions.

The loading amount of the α-Fe_2_O_3_-ZnO NR hybrid nanostructure on the working electrode of SPCE was optimized to obtain the optimum sensing response towards nitrite. The 4 µL of α-Fe_2_O_3_-ZnO NR hybrid nanostructure-modified electrodes yielded a better response towards nitrite. Different nitrite concentrations were tested with the α-Fe_2_O_3_-ZnO NR hybrid/SPCE sensor, and their DPV responses were recorded. The DPV response showed well-defined signals for each nitrite concentration (Figure 7a). A linear current response increase was noted, which is in direct proportion to the increasing nitrite concentrations (see inset of Figure 7a). However, at higher concentrations, the DPV response decreases, which may be due to the saturation of the active surface of the α-Fe_2_O_3_-ZnO NR hybrid-modified electrode. After measuring DPV responses three times, a linear corresponding calibration plot was plotted in Figure 7b. From the calibration plot, the nitrite sensor responded linearly up to the 400 µM nitrite concentration. The sensitivity of the α-Fe_2_O_3_-ZnO NR hybrid/SPCE sensor for nitrite was found to be 18.10 µA µM^−1^ cm^−2^, which was calculated by dividing the slope of the calibration curve (1.2856 µA/µM) with the geometrical area (0.071 cm^2^) of SPCE’s working electrode. The limit of detection (LoD) was found to be 0.16 µM (based on the signal and noise ratio of 3). The nitrite sensing performance metrics of the α-Fe_2_O_3_-ZnO NR hybrid have been benchmarked against contemporary nitrite sensors in Table 1. As shown in the Table, our sensor surpasses most of the previously reported sensors [49,50,51,52,53,54,55,56,57,58,59]. Principally, our sensor showed impressive LoD and sensitivity. From previous studies, the nitrite concentration in healthy human blood is in the range of 0.4–1.2 µM [60]. The wide linear range of this nitrite sensor can be useful to analyze nitrite levels in real samples directly, without diluting samples. The better performance of the fabricated sensor is credited to the combined advantages of α-Fe_2_O_3_ and ZnO NR. The α-Fe_2_O_3_ nanostructure, with its superior electrocatalytic properties, works in synergy with ZnO, which provides an excellent surface for modification and enhances the electrochemical signal. This synergistic interaction between the materials enhances the sensor’s overall performance, leveraging both the physical and electrochemical attributes of the α-Fe_2_O_3_-ZnO NR hybrid nanostructure.

### 3.4. Interference, Stability, and Reproducibility Studies

**Figure 8:**
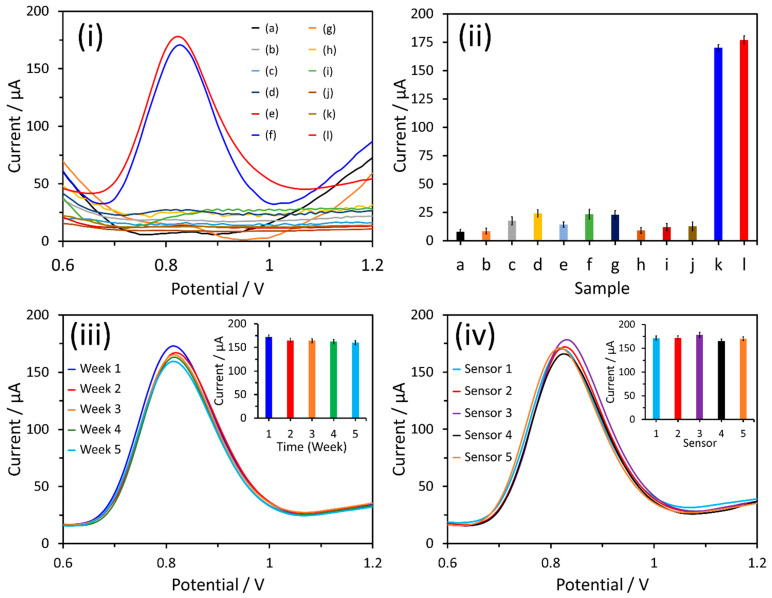
(**i**) DPV response of the α-Fe_2_O_3_-ZnO NR hybrid/SPCE sensor in PBS (a) and the presence of 100 μM interfering ions, i.e., (b) KCl, (c) NH_4_Cl, (d) CuSO_4_, (e) Pb(NO_3_)_2_, (f) NaNO_3_, (g) Na_2_SO_4_, (h) MgSO_4_, (i) CaCl_2_, (j) CaCO_3_, (k) nitrite, and (l) 100 μM nitrite, with each 100 μM interfering ion; (**ii**) histogram showing current response for interfering test, (**iii**) storage stability, and (**iv**) reproducibility tests of the fabricated sensors. Insets in (**ii**) and (**iii**) show the histograms of storage stability and reproducibility tests, respectively.

Selectivity stands as a crucial characteristic in the analytical efficacy of electrochemical sensors. To this end, the behavior of nitrite was monitored in the presence of various commonly encountered interfering ions (Figure 8i). The outcomes demonstrated that the electrochemical signal of nitrite remained stable and unaffected by the presence of 100 µM of KCl, NH_4_Cl, CuSO_4_, Pb(NO_3_)_2_, NaNO_3_, Na_2_SO_4_, MgSO_4_, CaCl_2_, and CaCO_3_ (Figure 8ii). The observed variation in the peak current did not exceed ±5%, showcasing satisfactory selectivity in the presence of interfering ions, highlighting the sensor’s adeptness in distinguishing nitrite from potential interfering ions.

To evaluate the storage stability of the α-Fe_2_O_3_-ZnO NR hybrid/SPCE sensors, they were kept at room temperature in ambient air, and their sensing capabilities were assessed over a period of five weeks. This was accomplished by testing their DPV response to 100 μM nitrite (Figure 8iii). Each test was conducted three times to ensure accuracy, and the results were presented as a histogram (see inset of Figure 8iii). Remarkably, after five weeks of storage under these conditions, the α-Fe_2_O_3_-ZnO NR hybrid/SPCE sensors retained approximately 92.6% of their original response. This high level of retention underscores the sensor electrode’s exceptional storage stability, demonstrating its robustness and reliability over extended periods without significant degradation in performance.

We finally assessed the reproducibility of the α-Fe_2_O_3_-ZnO NR hybrid/SPCE electrochemical sensor, and a series of tests were conducted using five identical α-Fe_2_O_3_-ZnO NR hybrid/SPCE sensors to measure the concentration of 100 µM nitrite (Figure 8iv). The consistency of the sensor’s performance was indicated by the relative standard deviation (RSD) of the peak currents, which was found to be 3.25% (inset, Figure 8iv). This low RSD value underscores the sensor’s reliable reproducibility, showcasing its ability to deliver consistent electrochemical responses across multiple devices under identical conditions. This attribute is crucial for ensuring the sensor’s practical applicability in real-world scenarios, where precision and reliability are paramount.

### 3.5. Nitrite Ion Quantification in Serum

**Figure 9:**
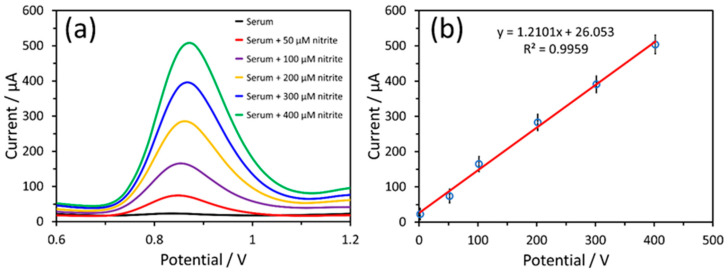
(**a**) DPV response of the α-Fe_2_O_3_-ZnO NR hybrid/SPCE sensor in human serum with increasing nitrite concentration and (**b**) calibration plot of the sensor.

The real-world applicability of the α-Fe_2_O_3_-ZnO NR hybrid/SPCE sensor for nitrite detection was investigated through DPV measurements conducted on human serum. This involved spiking the serum with different nitrite concentrations ranging from 50 μM to 400 μM, and measuring the DPV response with the same sensor, as illustrated in Figure 9a. Similar to the results obtained with spiked PBS, consistent DPV response peaks were noted, indicating the sensor’s effective sensing capability in complex biological matrices. To compare sensitivity, the slope of the calibration curve obtained from these DPV measurements was calculated (Figure 9b). The sensor sensitivity (17.05 µA µM^−1^ cm^−2^) in human serum was slightly less compared to PBS. However, these results are deemed satisfactory, especially when taking into account the intricate composition of human serum as a testing matrix. This approach provided a clear basis for evaluating the sensor’s performance in a biological environment.

Furthermore, the precision of the described α-Fe_2_O_3_-ZnO NR hybrid/SPCE sensor was verified by analyzing spiked samples through ion chromatography, and these findings are detailed in Table 2. The agreement between the two analytical approaches was determined by comparing the concentration of nitrite ions measured using the α-Fe_2_O_3_-ZnO NR hybrid/SPCE sensor to that obtained via ion chromatographic analysis. Remarkably, a satisfactory level of accordance, ranging from 95.4 to 100.9%, was achieved, showcasing a strong correlation between the two methodologies [61]. This underscores the robustness and reliability of the α-Fe_2_O_3_-ZnO NR hybrid/SPCE sensor system in quantifying nitrite ions, affirming its potential utility in analytical applications.

## 4. Conclusions

In this work, we have developed an α-Fe_2_O_3_-ZnO NR hybrid nanostructure using a two-step hydrothermal method. This simplified method enables the synthesis of large amounts of hybrid nanostructures, where the decoration of α-Fe_2_O_3_ NPs over the ZnO NR surface is swift. Comprehensive morphological and elemental analyses were conducted to verify the effective attachment of α-Fe_2_O_3_ NPs to the ZnO NR surface, confirming the successful and uniform modification. The α-Fe_2_O_3_-ZnO NR hybrid nanostructure was used to fabricate enzymeless nitrite sensors. The electrochemical properties of this material were evaluated by using it on the working electrode of SPCE in voltammetric tests. The α-Fe_2_O_3_-ZnO NR hybrid nanostructure-based nitrite sensor demonstrates an impressive electrocatalytic oxidation of nitrite. The results demonstrated its efficacy in detecting nitrite up to the 400 μM concentration with remarkably high sensitivity (18.10 µA µM^−1^ cm^−2^) and LoD (0.16 μM). Additionally, other excellent properties (i.e., selectivity, stability, and reproducibility) of the fabricated nitrite sensor open up possibilities for further exploration. Furthermore, the electrocatalytic performance of the nitrite sensor was tested in the serum sample, which resulted in slightly less sensitivity (17.05 µA µM^−1^ cm^−2^). This may be due to the complex nature of human serum as a testing matrix. The successful application of sensors in serum nitrite detection positions it as a promising candidate for nitrite detection. The precision of these sensors was verified by comparing their results with ion chromatographic analyses of spiked samples, revealing satisfactory results. The α-Fe_2_O_3_-ZnO NR hybrid nanostructure-based sensor holds promise for adaptation to modify it with enzymes or other metal/metal oxide nanostructures and expand its applicability across various analyte detection.

## Figures and Tables

**Table 1:** Nitrite sensing performance metrics of α-Fe_2_O_3_-ZnO NR hybrid/SPCE compared to previously reported nitrite sensors.

Nitrite Sensor	Detection Method	Sensitivity (µA µM^−1^ cm^−2^)	Linear Range (µM)	Detection Limit (µM)	Ref.
Pd-Pt/PGaN	Amperometry	0.15	1–300	0.95	[49]
TiO_2_-Ti_3_C_2_TX-CTAB-CS/GCE	DPV	-	3–250	0.85	[50]
Pd-Grp/GCE	Amperometry	0.29	0.3–50.7	0.071	[51]
Hb-Au NPs-TiO_2_/GCE	Amperometry	-	4–350	1.2	[52]
Ag-HNT-MoS_2_/SPCE	Amperometry	0.0899	2–425	0.7	[53]
Pd NPs-SWCNT/PET	DPV	0.417	2–238	0.25	[54]
Au NPs-MoS_2_-NSs/GCE	DPV	-	5–260	0.5	[55]
Au NPs-CS-MXene/GCE	Amperometry	0.5178	0.5–335.5	0.069	[56]
GO-PANI-Au NPs/GCE	Amperometry	-	0.5–240	0.17	[57]
La_2_CuO_4_ NPs/GCE	DPV	0.317	0.05–25	0.00262	[58]
MWCNT-CS/SPE	Amperometry	0.2044	Up to 1700	2.3	[59]
α-Fe_2_O_3_-ZnO NR/SPCE	DPV	18.10	0–400	0.16	This work

Pd: palladium; Pt: platinum; PGaN: porous gallium nitride; TiO_2_: titanium dioxide; CTAB: hexadecyl trimethyl ammonium bromide; CS: chitosan; GCE: glassy carbon electrode; Grp: graphite; Hb: haemoglobin; Au: gold; NPs: nanoparticles; Ag: silver; HNT: halloysite nanotube; MoS_2_: molybdenum disulphide; SWCNT: single-walled carbon nanotube; PET: polyethylene terephthalate; NSs: nanosheets; GO: graphene oxide; PANI: polyaniline; La_2_CuO_4_: lanthanum copper oxide; MWCNT: multi-walled carbon nanotube.

**Table 2:** Nitrite ion quantification in serum sample (n = 3).

Sample	Nitrite Concentration (μM)	Nitrite Added (μM)	Detection with DPV Method (μM)	Detection with Ion Chromatography (μM)	Accordance (%) *
Human serum	1.0	0	0.96	1.0	96
	1.0	50	50.2	50.8	98.8
	1.0	100	101.5	100.6	100.9
	1.0	200	192	198	96.9
	1.0	300	289	296	97.6
	1.0	400	374	392	95.4

* $Accordance\;(\%)=\frac{DPV\;method\;(\mu M)}{Ion\;chromatography\;(\mu M)}\times 100$.

## Data Availability

Data are contained within the article.
